# Vitamin C Protected Human Retinal Pigmented Epithelium from Oxidant Injury Depending on Regulating SIRT1

**DOI:** 10.1155/2014/750634

**Published:** 2014-07-22

**Authors:** Wei Wei, Langen Li, Yufeng Zhang, Geriletu, Jia Yang, Yanmei Zhang, Yiqiao Xing

**Affiliations:** ^1^Department of Ophthalmology, Inner Mongolia People's Hospital, 20 Zhaowuda Road, Hohhot, Inner Mongolia 010017, China; ^2^Department of Neurology, the Affiliated Hospital of Inner Mongolia Medical University, 1 Tongdao North Road, Hohhot, Inner Mongolia 010050, China; ^3^Department of Neurology, Inner Mongolia People's Hospital, 20 Zhaowuda Road, Hohhot, Inner Mongolia 010017, China; ^4^Department of Ophthalmology, Renmin Hospital of Wuhan University, 99 Zhangzhidong Road, Wuchang, Wuhan 430060, China

## Abstract

The purpose was to investigate the protective effects of Vitamin C (Vit C) and the regulatory mechanism between Vit C and sirtuin 1 (SIRT1) in PREs during oxidative stress as Vit C and SIRT1 exerted famous effects as antioxidants. We found that moderate Vit C (100 *µ*M) prevented ARPE-19 cells from damages induced by H_2_O_2_, including increasing viability, reducing apoptosis, and attenuating intracellular ROS levels. But lower and higher concentration of Vit C had no effects. Further results indicated that Vit C caused the dysregulation of some stress responses factors (SIRT1, p53 and FOXO3) in ARPE-19 cells response to H_2_O_2_. Moreover we found that SIRT1 activator resveratrol (SRV) stimulated significantly the protective effects of moderate Vit C, provided the property of antioxidative stress for the lower and higher concentration of Vit C in ARPE-19 cells as well. Consistently, nicotinamide (NA) relieved the protective functions of moderate Vit C. Interestingly, data also revealed the dysregulation of p53 and FOXO3 was dependent on the regulation of SIRT1 rather than Vit C. Summarily, the protective effect of Vit C against oxidative stress was involved in regulation of SIRT1. It suggested that combined application of Vit C and RSV might be a promising therapeutic method for AMD.

## 1. Introduction

Age-related macular degeneration (AMD) is the most common cause of vision loss in the elderly [1, 2]. AMD patients have extensive free radicals, the injury of the proteins, lipids, DNA, and mitochondria, in their retinal pigment epithelial cells (RPEs) by postmortem [3]. Proteomic analysis also found numerous proteins which were caused by oxidative damage in drusen [4]. Some reports have found that antioxidants could slow the process of AMD [5]. Thereby oxidative stress plays an important role in AMD pathogenesis and oxidative injury contributed to the pathogenesis of AMD [1, 6]. RPEs are essential for vision by providing nutrients as transporters and maintenance functions for photoreceptors [7]. One of the potential contributors to AMD pathology is dysfunction of RPEs [8]. So, finding effective therapeutics that protect RPEs from damage is promising treatments for AMD.

Oxidative stress promoted the development of age-related RPEs degeneration, dysfunction, and loss [9, 10]. Cells have some protective strategies to minimize oxidative damage. The most famous defense system is to develop the endogenous antioxidants, including superoxide dismutases (SODs), carotenoids, and vitamins [6]. Vitamin C (Vit C), known for its role in development and maintenance of connective tissues, has effects on bone formation, wound healing, and the maintenance of healthy gums. The famous roles of Vit C are protection of immune system, reduction of allergic reactions, and combating infections [11]. It is also an antioxidant that protects the body against oxidative stress. Vit C has been regarded as the most important one which provides protection against atherogenesis and Alzheimer's disease [11, 12]. In recent studies, Vit C attenuated the oxidative stress in diabetic aged rats [13, 14]. Regarding the role of Vit C in retinopathy, Vit C and Vit E provided protection for retinal glutathione reductase, glutathione peroxidase, and superoxide dismutase activities. So, Vit C and Vit E relieve retinopathy induced by oxidative stress [14]. Nevertheless, Vit C did not mitigate the effects of oxidative stress on RPEs [15]. In the study of Zeitz et al., Vit C, potent hydroxyl radical quenchers* in vitro*, failed to protect cultured ARPE-19 cells from oxidative stress induced cell death [16]. Interestingly, Yin et al. reported that Vit C supplementation of 100–200 *μ*M appeared to strongly inhibit oxidative stress, but no additional advantage was found as the concentration of Vit C was higher [17]. So, Vit C, the wildly used antioxidant, which exerted performances in protection of RPEs against oxidative stress continued to merit further investigation.

SIRT1, a class III protein deacetylase, played regulatory roles in cellular stressors, genotoxic, oxidative, and proteotoxic stress [18, 19]. It was found that moderate overexpression of SIRT1 provided protection for mouse cardiac muscle against oxidative stress [20]. SIRT1 could also prohibit some transcription factors, which regulated cellular redox balance such as inhibition of the transactivation capacity of NRF2 in HepG2 cells [21]. However we kept enhancing understanding on detailed mechanism of the SIRT1 against oxidative stress, especially in RPEs.

In this study, we focused on the antioxidant effects of Vit C and the regulatory mechanism between Vit C and SIRT1 in PREs during oxidative stress.

## 2. Materials and Methods

### 2.1. Cell Culture

ARPE-19 cell line (human RPEs) was purchased from American Type Culture Collection (ATCC, Manassas, VA, USA). Cells were cultured in Dulbecco's modified Eagle medium (DMEM) containing 10% heat-inactivated fetal bovine serum (FBS) (Gibco, Invitrogen, Carlsbad, USA), 100 *μ*g/mL of streptomycin, and 100 U/mL of penicillin in 5% CO_2_ at 37°C. The cells were subcultured when reaching about 90% confluence.

### 2.2. Cells Viability and Apoptosis Assay

ARPE-19 cells were grown at 80% confluence. Then cells were treated with H_2_O_2_ (100 *μ*M) for 12 h or 24 h after supplementation of Vit C as indicated concentration. Cell viability was assayed by a 3-(4, 5-dimethylthiazol-2-yl)-2, 5-diphenyltetrazolium bromide (MTT) assay (Sigma, St. Louis, MO, USA) after being treated with indicated experiments. Briefly MTT was added to the cells at 37°C for 4 h. After incubation, MTT-containing medium was discarded and dimethyl sulfoxide (DMSO) was performed to dissolve formazan crystals. Optical densities (OD) were measured at 450 nm by VersaMax microplate reader (Molecular Devices, Sunnyvale, CA, USA). Viability was normalized by OD value/cell number and ARPE-19 cells without treatment (control) were denoted as 100%. Apoptosis was assessed by annexin V-FITC apoptosis detection kit (Sigma-Aldrich, St. Louis, Missouri) following the manufacturer's protocol.

### 2.3. Measurement of Intracellular ROS Levels

Intracellular ROS levels were measured by using intracellular ROS assay kit (Cell Biolabs, San Diego, CA). Briefly, ARPE-19 cells were incubated with 2′,7′-dichlorodihydrofluorescein diacetate (DCFH-DA) in the culture medium for 30 min at 37°C. The cells were assessed by using flow cytometry at 488 and 525 nm wavelengths. DCFH fluorescence of the cell lysate was captured and quantified by ImageJ software (NIH). The average fluorescence intensity was analyzed from five fields for each treatment.

### 2.4. Cell Treatments

Cells were incubated with Vit C (SIGMA-Aldrich) at indicated concentration; then H_2_O_2_ (100 *μ*M) was added for 12 h or 24 h. Then, H_2_O_2_ was detoxified by adding catalase (100,000 *μ*/L; Worthington Biochemical) at the end of the incubation [22]. 10 mM resveratrol (RSV) and 5 mM nicotinamide (NA) (Sigma, St. Louis, USA) were performed to incubate cells before treatments of oxidants [23]. SIRT1 knockdown was performed by using siRNA for SIRT1 and shRNAi MISSION nontarget shRNA Control/Puro was performed as negative control (SIRT1si and C-SIRT1si) (Santa Cruz Biotechnology, Santa Cruz, CA, USA). pRC/CMV-SRIT1 and pcRC/CMV (Promega, Madison, WI) expression vectors (SIRT1up and C-SIRT1up) were acquired from Promega (Promega, Madison, WI). Cell transfection was conducted by using Lipofectamine 2000 reagent (Invitrogen, Carlsbad, CA, USA) according to the manufacturer's instructions.

### 2.5. RNA Extraction and Quantitative Real-Time Polymerase Chain Reaction (qRT-PCR)

Total RNA was extracted by using RNeasy kits (Qiagen, Valencia, CA). First-strand cDNA was synthesized using SuperScript III (Invitrogen). qRT-PCR was performed on ABI 7500 with SYBR Premix Ex Taq kit (Takara, Japan). GAPDH was denoted as the internal control. Data were normalized by using 2^−ΔΔCt^ method as relative quantification. The used primers were p53: F agt cac agc aca tga cgg agg t, R tac aca tgt act tgt agt gga t; FOXO3: F aac aga cca gcc acc ttc tct t, R gct gac aga att tga caa ggc a; SirT1: F tgt ggt aga gct tgc att gat ctt, R ggc ctg ttg ctc tcc tca t; GAPDH: F gga gtc aac gga ttt ggt c, R gga atc att gga aca tgt aaa c. The reactions were started at 95°C for 5 min followed by 40 cycles of 95°C for 15 sec and 60°C for 30 sec and extension for 1 min at 72°C.

### 2.6. Western Blot

Proteins were extracted from cells. Then, the protein samples were subjected to 10% SDSPAGE. Proteins were transferred onto nitrocellulose membranes after electrophoresis. The membrane was blocked with TBST (50 mM Tris-HCl, pH 7.5, 150 mM NaCl, and 0.05% Tween 20) containing 5% nonfat milk or 5% BSA for 30 min. The membrane was incubated with primary antibodies overnight at 4°C. Then, goat anti-mouse IgG or goat anti-rabbit IgG (Pierce) was utilized to visualize the results, which ECL detection systems (Super Signal West Femto, Pierce) were conducted to assay. Human monoclonal primary antibodies were used as follows: anti-FOXO3 (Cell Signaling, Beverly, MA), anti-p53 (Cell Signaling, Beverly, MA), SIRT1 primary antibody (Sigma-Aldrich), and *β*-actin antibody (Sigma-Aldrich).

### 2.7. Statistical Analysis

Data were denoted as means ± standard deviation (SD). Student's* t*-test was used to analyze differences of two groups. The experimental results were analyzed with one-way ANOVA to compare the differences of three groups (or more than three). The criterion for significance was *P* < 0.05. Statistical analyses were conducted by SPSS 17.0 software (SPSS, Chicago, IL, USA). The criterion for significance was expressed as *P < 0.05 and **P < 0.01. All the experiments were performed in triplicate.

## 3. Results

### 3.1. Moderate Vit C Prevented RPEs from Oxidant Injury Induced by H_2_O_2_

**Figure 1:**
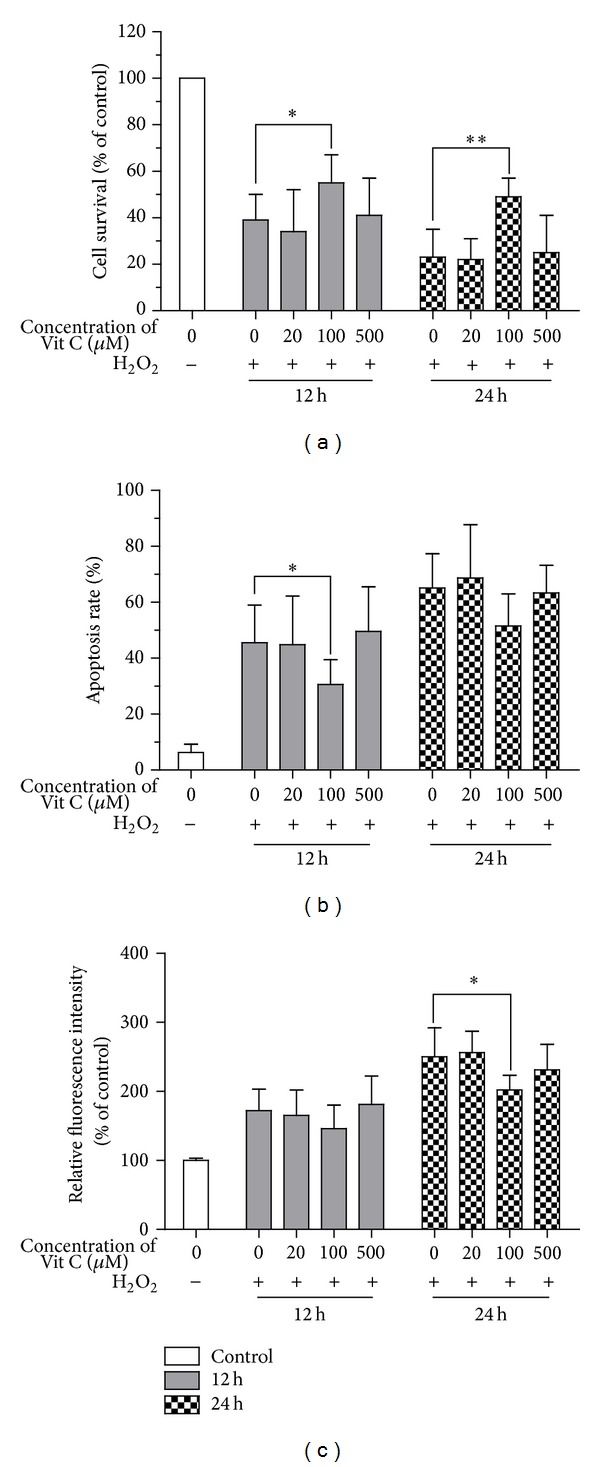
Effects of Vit C on viability, apoptosis, and intracellular ROS of ARPE-19 cells induced by H_2_O_2_. ARPE-19 cells were incubated for 24 h with 0 *μ*M, 20 *μ*M, 100 *μ*M, or 500 *μ*M of Vit C and then exposed to 100 *μ*M of H_2_O_2_ for 12 h or 24 h. Viability, apoptosis, and intracellular ROS were measured by using MTT assays, TUNEL assays, and DCFH-DA, respectively, as shown in Section 2. Normal ARPE-19 cells which did not undergo treatment were expressed as “control.” Data were expressed as a percentage of cell survival (a), apoptosis (b), and relative fluorescence intensity (c). Bars represented mean ± SD of three independent experiments. ANOVA was performed to analyze the differences statistically. Statistical significance was expressed as *P < 0.05 and **P < 0.01.

To investigate the effect of Vit C on oxidant injury induced by H_2_O_2_, the ARPE-19 cells were pretreated with Vit C at 0 *μ*M, 20 *μ*M, 100 *μ*M, or 500 *μ*M. Then, the RPEs were incubated with H_2_O_2_ at 100 *μ*M for 12 h and 24 h. We first detected the effect of the Vit C on viability during oxidative stress in ARPE-19 cells. There was significant difference in viability when the concentration of Vit C was increased to 100 *μ*M (*P* < 0.05 at 12 h and 24 h). Higher concentration (500 *μ*M) and lower concentration (20 *μ*M) of Vit C could not significantly influence the cell viability in the presence of H_2_O_2_ (Figure 1(a)). Corresponding to the effect of Vit C on viability, Vit C reduced the number of apoptotic RPEs significantly at 100 *μ*M concentration (*P* < 0.05) after 12 h exposure to H_2_O_2_ Figure 1(b). Because of accumulation of intracellular ROS in AMD [24], we also tested the intracellular ROS.  Figure 1(c) displayed the fact that treatment with Vit C caused the changes of intracellular production of ROS also, especially when the concentration of Vit C reached 100 *μ*M after 24 h exposure to H_2_O_2_ (*P* < 0.05). These findings illustrated that it was moderate Vit C (100 *μ*M) that had protective effect against oxidant injury induced by H_2_O_2_ in RPEs.

### 3.2. Vit C Affected the Expression of the SIRT1 Transcription Factor and Stress Responses Factors (p53 and FOXO3)

**Figure 2:**
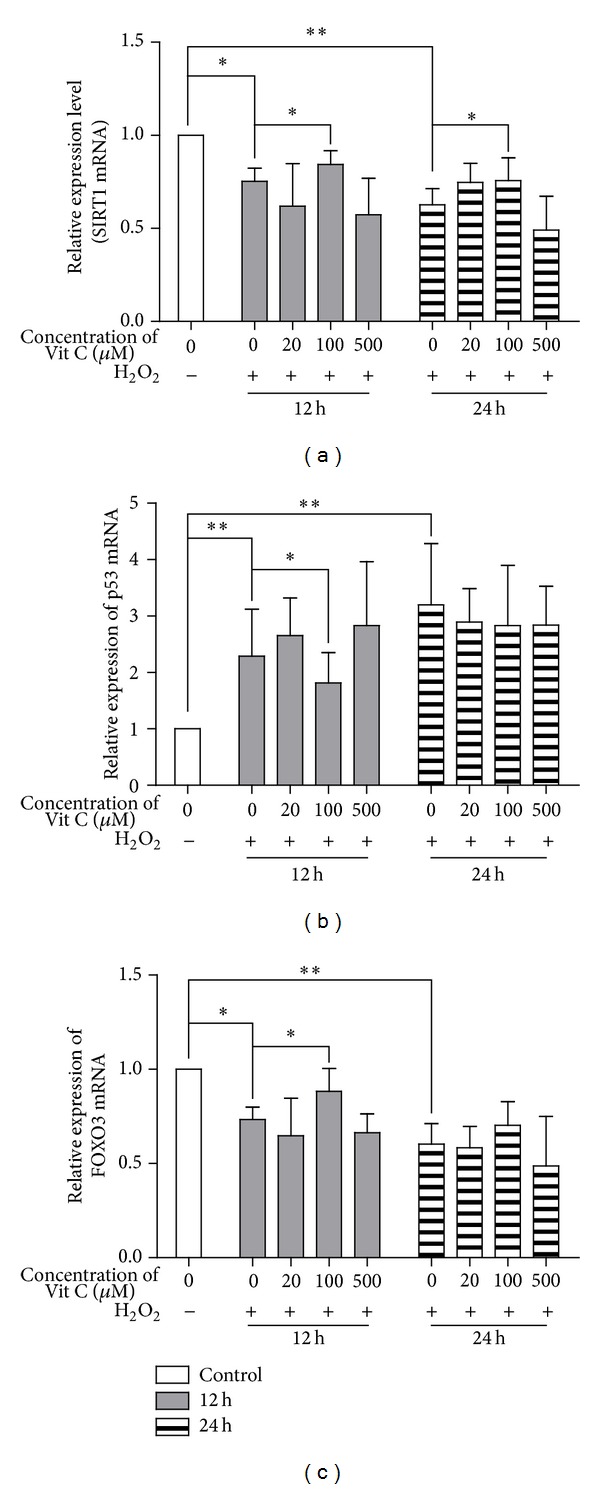
Vit C resulted in the dysregulation of SIRT1, p53, and FOXO3 during oxidative stress in ARPE-19 cells. ARPE-19 cells were treated with indicated methods. The expression of SIRT1 (a), p53 (b), and FOXO3 (c) mRNA was detected by qRT-PCR. Ct values were normalized by 2^−ΔΔCt^ method as relative quantification. Bars represented mean ± SD of three independent experiments. ANOVA was performed to analyze the differences statistically. Statistical significance was expressed as *P < 0.05 and **P < 0.01.

As the key and ubiquitous roles of SIRT1 in inflammaging and senescence, especially in antioxidative stress, we suspected if SIRT1 referred to the mechanism of protection of Vit C against oxidative stress [25]. To confirm the hypothesis, we assayed the expression of SIRT1 when RPEs were treated with Vit C after 12 h or 24 h exposure to H_2_O_2_. The relative expression of SIRT1 was shown in Figure 2(a). The results showed that H_2_O_2_ reduced the expression of SIRT1 and Vit C affected the expression of SIRT1. The expression of SIRT1 reached significant difference when the level of Vit C concentration was 100 *μ*M (*P* = 0.021 and *P* = 0.039 at 12 h and 24 h, resp.). We also detected the expression of stress responses factors (p53 and FOXO3) to further support our hypothesis.  Figure 2(b) displayed the fact that the expression of p53 was upregulated significantly when the RPEs were pretreated with moderate Vit C (100 *μ*M) after exposure to H_2_O_2_ for 12 h (*P* = 0.046). The expression of FOXO3 gene was increased in accordance with SIRT1, significantly at 12 h with 100 *μ*M Vit C (*P* = 0.032) (Figure 2(c)). These findings revealed that only the moderate Vit C (100 *μ*M) regulated the SIRT1 transcription factor and stress responses factors (p53 and FOXO3). It is also suggested that if the protective effect of Vit C against oxidative stress was involved in a SIRT1 signaling pathway because of the regulatory effects of SIRT1 on p53 and FOXO3.

### 3.3. The Protective Effect of Vit C against Oxidative Stress Was Involved in Regulation of SIRT1

**Figure 3:**
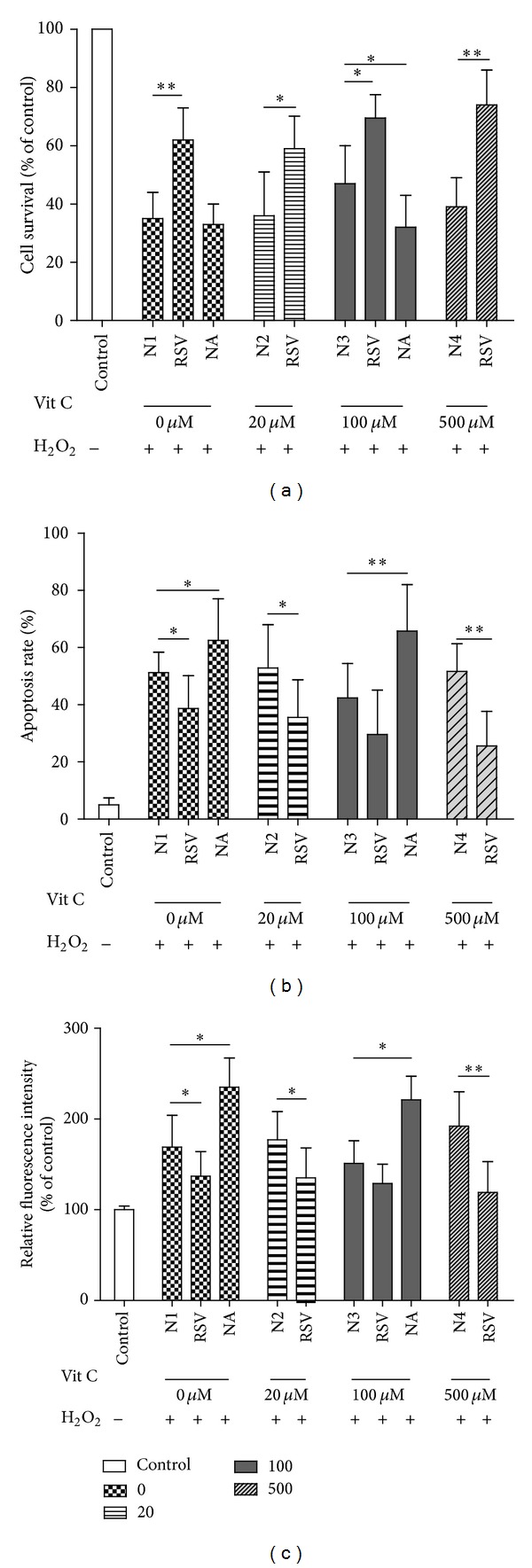
SIRT1 changed the effects of Vit C on viability, apoptosis, and intracellular ROS of ARPE-19 cells induced by H_2_O_2_. ARPE-19 cells were preincubated with RSV (10 mM) or NA (5 mM) for 4 h. Then, cells were exposed to H_2_O_2_ for 12 h after treatments of indicated concentration of Vit C. Viability (a), apoptosis (b), and intracellular ROS (c) were measured and compared among different groups. N1, N2, N3, and N4 were standing for the cells treated with H_2_O_2_ and without Vit C preincubation, cells treated with H_2_O_2_ and 20 *μ*M Vit C, cells treated with H_2_O_2_ and 100 *μ*M Vit C, and cells treated with H_2_O_2_ and 500 *μ*M Vit C, respectively. Single treatment was performed in triplicate. Statistical significance was expressed as *P < 0.05 and **P < 0.01.

To reveal the crosstalk between Vit C and SIRT1 during oxidative stress in ARPE-19 cells, we analyzed viability, apoptosis, and intracellular ROS after treatments of Vit C at indicated concentration and treatments of SIRT1 activator RSV or SIRT1 inhibitor NA upon 12 h exposure to H_2_O_2_ as shown in Figure 3. The results of the MTT assays showed that the RSV could significantly augment the promoting effects of Vit C on the cells viability, compared to the N group (N1, N2, N3, and N4). Importantly, RSV increased the effects of lower Vit C (20 *μ*M) and higher Vit C (500 *μ*M) on cells viability in the presence of  H_2_O_2_ also, whereas inhibitor NA could attenuate the viability dramatically when the concentration of Vit C was 100 *μ*M (*P* < 0.05) as shown in Figure 3(a). TUNEL assays displayed the fact that RSV not only stimulated the effects of moderate Vit C (100 *μ*M) on reducing the number of apoptotic cells but also provided the antiapoptotic ability for the lower Vit C (20 *μ*M) and higher Vit C (500 *μ*M) significantly (*P* < 0.05). NA increased the number of apoptotic cells which were pretreated with moderate Vit C (100 *μ*M) significantly, compared with the N group (N1, N2, N3, and N4) (Figure 3(b)). It meant that RSV could promote the antiapoptotic effects of Vit C, and NA attenuated the antiapoptotic effects of Vit C during oxidative stress. Treatment with RSV and NA resulted in inhibition and promotion of intracellular production of ROS also as shown in (Figure 3(c)). All findings illustrated that SIRT1 played key roles in the protective effect of Vit C against oxidative stress.

**Figure 4:**
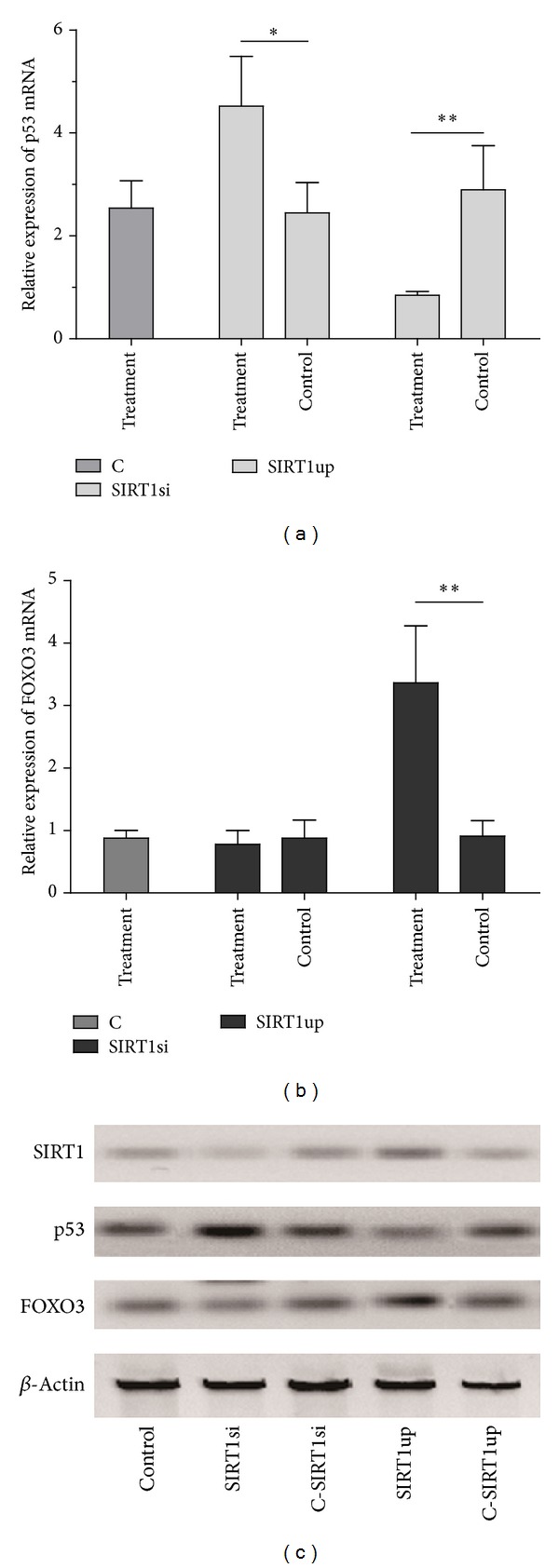
Regulation of FoxO3 and p53 was involved in the expression of SIRT1 rather than Vit C. ARPE-19 cells underwent SIRT1 knockdown and overexpression of SIRT1 by using siRNA (SIRT1si group), pRC/CMV-SRIT1 (SIRT1up group), and its control (C-SIRT1si and C-SIRT1up groups, resp.). Then, cells were exposed to 100 *μ*M of H_2_O_2_ for 12 h after being incubated for 24 h with 100 *μ*M of Vit C. The expression of p53 (a) and FOXO3 (b) mRNA was detected by qRT-PCR and was normalized by 2^−ΔΔCt^ method as relative quantification. The expression of SIRT1, p53, and FOXO3 proteins was assayed by western blot in ARPE-19 cells with indicated treatments (c). Bars represented mean ± SD of three independent experiments. ANOVA was performed to analyze the differences statistically. Statisticalsignificance was expressed as *P < 0.05 and **P < 0.01.

To exclude the possibility that Vit C contributed to the expression of stress responses factors (p53 and FOXO3) which have protective effects on apoptosis, viability, and intracellular ROS, we examined the expression of p53 and FOXO3 after knockdown or overexpression of SIRT1 genes in RPEs with supplementation of 100 *μ*M Vit C during oxidative stress. The comparisons of p53 expression among indicated groups found SIRT1 knockdown and upregulated SIRT1 significantly increased and inhibited the expression of p53, respectively (Figure 4(a)). It was found that FOXO3 was decreased slightly in SIRT1 silenced RPEs and the upregulated SIRT1 promoted the expression of FOXO3 significantly as shown in Figure 4(b).  Figure 4(c) displayed the expression of SIRT1, p53, and FOXO3 proteins in indicated groups. It further supported the fact that SIRT1 was the key regulator to modulate the p53 and FOXO3. These findings indicated that the regulatory effects of moderate Vit C on the expression of FOXO3 and p53 were closely related to the SIRT1. In other words, the protective effect of Vit C against oxidative stress was involved in regulation of SIRT1.

## 4. Discussion

Oxidative stress has been considered as a major factor to contribute to aging associated diseases, including Alzheimer's disease, diabetes mellitus, cardiovascular disorders, and AMD [6, 12, 14]. Vitamins have primary effects on oxidative stress as antioxidants. Vit C provided protection against atherogenesis and Alzheimer's disease as antioxidants, except for protection of immune system, reduction of allergic reactions and combating infections [11]. In terms of protection of retina, Vit C could relieve retinopathy induced by oxidative stress in rats and played controversial roles in preventing human RPEs from oxidative stress [13]. Owing to extensive clinical application of Vit C, the investigation of effects of Vit C on RPEs in response to oxidative stress is a challenging and promising therapeutic method for protection of RPEs to delay the process of AMD [26].

In this paper, we illustrated that the injuries of RPEs induced by H_2_O_2_ were not reduced dramatically by Vit C treatment at lower and higher concentration. It was Vit C supplementation of 100 *μ*M that appeared to significantly alleviate oxidative stress, concluding viability, apoptosis, and intracellular ROS (Figure 1). The data were similar to the results of Yin et al. about the functions of Vit C on RPEs [17]. Our findings further illustrated the effects of Vit C on viability, apoptosis, and intracellular ROS of RPEs response to oxidative stress. Even though the beneficial action of Vit C against oxidative stress was well reported in the literature, the advantage of Vit C was too obvious to exert antioxidative effects on RPEs as a matter of fact.

Some authors had reported that Vit C modulated the level of SIRT1 and played an important roles in lung development through affecting oxidant-antioxidant balance in rats [27]. Vit C also stimulated the activity of SIRT1 which deacetylated 7-amino-4-methylcoumarin-labeled acetylated peptide [28]. Conversely, recent reports had documented pretreatment human dermal fibroblasts cells with 2-O-*α*-glucopyranosyl-l-ascorbic acid (2A-Vit C) before H_2_O_2_ exposure significantly inhibited this decrease in SIRT1 expression, whereas ascorbic acid (Vit C) had no effect [29]. So we made a hypothesis that crosstalk between Vit C and SIRT1 might be highly related to microenvironment. And the mechanism needs to merit further investigation. For this reason, we surveyed to characterize the crosstalk between Vit C and SIRT1 in RPEs during oxidative stress. In this paper, we found that Vit C treatments affected the level of SIRT1 in RPEs under H_2_O_2_-mediated stress. Interestingly, the increase of SIRT1 level with consequent enhancement of protection PREs from damage induced by H_2_O_2_ occurred when concentration of Vit C was 100 *μ*M (Figure 2(a)). The results implied that the benefit of Vit C action might closely be related to the expression level of SIRT1. SIRT1 modulates lots of signaling pathways concerned with cellular senescence, proliferation, and apoptosis, because of its ability to deacetylate some cytokines such as forkhead box class O (FOXO)3, NF-*κ*B, and p53 [30, 31]. In this paper, levels of SIRT1 associated cytokines (p53 and FOXO3) displayed similar trend to the SIRT1 levels after Vit C treatments during oxidative stress (Figures 2(b) and 2(c)). These data revealed that Vit C could affect the expression of the SIRT1 transcription factors and SIRT1 associated cytokines.

In order to identify the relationship between SIRT1 and Vit C, we investigated if SIRT1 activator or inhibitor (RSV or NA) could affect the effect of Vit C on RPEs in the presence of oxidative stress. The results showed that RSV treatment significantly provided the ability against oxidative stress for the lower concentration of Vit C and higher concentration of Vit C. RSV also dramatically augmented the ability of moderate Vit C to prevent damage induced by H_2_O_2_. Furthermore, NA counteracted the protective roles of moderate level of Vit C (Figure 3).

Since p53 expression, which is regulated by SIRT1, could be modulated by Vit C in cancer and in alcoholic liver fibrosis also [28, 29], we investigated whether the SIRT1 or Vit C took the primary regulatory effects on stress responses factors (FOXO3 and p53) in PREs exposure to H_2_O_2_. Here, data displayed the fact that Vit C could not influence the expression of FoxO3 and p53 in the absence of SIRT1 (Figure 4). It confirmed that the SIRT1 caused the dysregulation of FoxO3 and p53 rather than Vit C in RPEs.

In conclusion, Vit C protected RPEs from oxidant injury depending on regulating SIRT1. This mechanism further suggested that combined application of Vit C and RSV, which exerted effective resistance to the damage of RPEs induced by oxidative stress, might be a promising therapeutic method for AMD.
